# Patients’ Perception of Recovery after Dental Implant Placement

**DOI:** 10.3390/medicina57101111

**Published:** 2021-10-15

**Authors:** Adrian Kahn, Daya Masri, Tamir Shalev, Haya Meir, Alon Sebaoun, Liat Chaushu

**Affiliations:** 1 Department of Oral and Maxillofacial Surgery, The Maurice and Gabriela Goldschleger School of Dental Medicine, Tel Aviv University, Tel Aviv 6997801, Israel; dr.adykahn@gmail.com; 2 Rabin Medical Center, Petach-Tikva 4941492, Israel; 3 Department of Oral and Maxillofacial Surgery, Rabin Medical Center, Petach-Tikva 4941492, Israel; dr.dayamasri@gmail.com; 4 Department of Periodontology and Oral Implantology, The Maurice and Gabriela Goldschleger School of Dental Medicine, Tel Aviv University, Tel Aviv 6997801, Israel; tamirshalev86@gmail.com (T.S.); hayameir@012.net.il (H.M.); alon.sebaoun@gmail.com (A.S.)

**Keywords:** dental implants, HRQOL, swelling, pain, analgesics

## Abstract

*Background and Objectives*: The success rates of surgical dental implant insertions are high. However, knowledge of patients’ recovery is still lacking. “Health-related quality of life” (HRQOL) questionnaires are gaining popularity in all fields of medicine. The present survey assessed the perception of recovery after the surgical placement of dental implants. *Materials and Methods*: Forty individuals (26 women and 14 men; mean age, 55 ± 12 years) filled a questionnaire evaluating patients’ perception of recovery for 7 consecutive days post-surgery. Confounding factors included age, gender, oral habits, smoking, bruxism, bone quality (tactile evaluation) and quantity, implant location, number of implants, implant type, length and diameter, one-stage vs. two-stage, and the need for bone grafting. *Results*: The most serious difficulties were found in swelling, which became minimal after 5 days, followed by eating everyday food, ability to enjoy everyday food, maximal pain and average pain (3 days); analgesics consumption (2.5 days); limitations in daily routine, mouth opening, and speech (2 days); swallowing and sleep (1.5 days); and, within 1 day, all other measures attained minimal levels. Gender, and implant location (anterior vs. posterior) were significant predictor variables exerting their different characteristic delayed recoveries. *Conclusions*: (1) Patients should expect, in general, recovery within 4 days after dental implant placement; (2) women will experience a delayed recovery, (3) implants placed in the intercanine area will result in postoperative eating difficulties for nearly one week, and (4) the number of implants placed during the same appointment has no effect on post treatment recovery.

## 1. Introduction

Endosseous dental implants have become an important method of treatment of complete or partial edentulism [1,2,3]. Complete restorations, overdentures, partial fixed/removable restorations, or even single tooth restorations are appropriate modes of prosthetic restoration using implants [1]. Dental implants have become successful since the development of designs and implantation procedures that result in a direct bone-implant interface without intervening fibrous tissue, detectable at the light microscope level [4]. This attachment of bone to the implant has been termed “osseointegration” and today is the goal of implant dentistry. Such an interface may be stable for many years. However, it does not mimic the attachment of natural teeth to the jawbone, as there is no periodontal ligament connecting the implant to the bone. Instead, the implant is ankylosed to the bone, a relationship that provides a tight rigid junction which can function effectively as a tooth replacement [4].

The advantages of the Brånemark protocol for achieving osseointegration have been demonstrated over many years of successful application [5]. Nevertheless, implant-supported rehabilitation is not always the best solution. Mechanical and biological complications do occur. The average survival of a dental implant is 8–10 years [6].

The relatively long healing time, during which the patient is subjected to significant inconvenience and difficulty in eating, as well as the requirement for two surgical procedures with associated expense, pain, and further inconvenience, are significant disadvantages and are often powerful disincentives to a treatment that would have great benefits to the patient’s health and quality of life [7,8,9,10,11,12,13]. 

Our knowledge of patients’ recovery is scarce [14]. “Health-related quality of life” (HRQOL) questionnaires are gaining popularity in all fields of medicine [14,15,16,17,18,19,20,21,22,23,24]. Fear of dental treatment has been ranked high by the population. Drilling, injection, and surgery are the most feared procedures. Fear and pain reflect actual experience, but pain perception is even more important. Managing and controlling information can help to prepare for treatment and eventually reduce pain. As dental implants grow in popularity, limited information is available on pain-associated implant insertion [25]. The present survey assessed the perception of recovery after the surgical placement of dental implants. 

## 2. Materials and Methods

The study was approved by the ethics committee of the Rabin Medical Center, Campus Beilinson, Israel (0674-19rmc). The HRQOL instrument (Appendix A) was previously described [14,23]. The questionnaire was never tested psychometrically, but it has been used successfully in numerous studies [14,23]. Consecutive patients who had been scheduled for the placement of dental implants were asked to enroll in a postoperative survey. All procedures were thoroughly explained to the patients, who signed an informed consent form. Only one maxillofacial surgeon (A.K.) inserted all the implants included in this study to avoid bias as a result of different operators. On the day of the surgery, after consenting to participate in the study, baseline data of the participants (age, gender, surgeon’s name, etc.) were recorded. The surgery was performed according to a standard protocol. Surgery commenced with local anesthesia containing a vasoconstrictor 1:100,000. Preparation of the implant site was performed with a low-speed contra-angle handpiece, with continuous cooling with sterile saline. The initial bone excavation was performed with a round bur, evaluating the density and thickness of the bone on the crest of the ridge. A bur was used to remove the top of the ridge until it reached sufficient width. A pilot drill was used to continue the bone preparation. Tactile sense was used to determine bone density [26]. Angulation was verified via parallel pin. The implant site preparation was continued as described in the surgical manual for each implant, with continuous saline cooling and occasional flushing of the site. All drilling was carried out with sufficient hand pressure to proceed at least 0.5 to 1 mm in drilling depth every five seconds, using pumping movements to facilitate access of the cooling fluid to the drilling site. The cutting surface of the bur did not contact bone for more than five out of every eight seconds. The site was thoroughly flushed with sterile saline. For screw-type implants placed in type I bone [26], the thread was cut in the bone with a tap before inserting the implant. Before implant placement, the recipient site was thoroughly flushed with sterile saline. The implant was placed with the implant/abutment junction at the crest of the bone, whenever possible. After insertion, the implant exhibited initial stability. The surgical procedure was recorded. Postoperative care included the following: no brushing or gentle brushing of the operated site for 2–3 weeks, rinsing with chlorhexidine mouthwash used 3 times daily for 60 s, liquid to soft diet, analgesics (Etodolac 400 mg, up to 3 per day per request) required for pain control, and antibiotic coverage. The patients were instructed to call immediately if any unusual signs or symptoms occurred.

After surgery, the HRQOL questionnaire was given to the patients. A daily telephone confirmed patient compliance. Individuals not responding to the questionnaire were excluded.

A visual analog scale (VAS) was used for pain assessment. A 5-point scale was used for other parameters [14,23]. Recovery was defined as ≤3 for pain and ≤2 for other parameters. The effect of confounding factors (age, gender, oral habits, smoking, bruxism, bone quality (tactile definition through drilling) and volume, implant location, number of implants, implant type, length and diameter, one- vs. two-stage surgery, and the need for bone grafting) on the recovery time were also assessed. The statistical significance was verified by a multiple-comparisons statistical analysis using the Fisher exact test, with *p* < 0.05 taken as the minimum criterion of significance.

## 3. Results

This study included 40 patients (26 women and 14 men; mean age, 55 ± 12 years). Ninety-eight implants were inserted in the 40 individuals, resulting in an average of 2.45 ± 1.43 (range: 1–6) implants per patient. Implant length averaged 12.27 ± 1.22 mm (range: 10–13 mm) and implant diameter averaged 4 ± 0.54 mm (range 3.3–5 mm). All the implants were from Zimmer Dental.

Bone quality [26] was type I in 5 (12.5%) cases, type II in 21 (52.5%) cases, and type III in 14 (35%) cases. Bone quantity [26] was type A in 12 (30%) cases, type B in 12 (30%) cases, type C in 15 (37.5%) cases, and type D in 1 (2.5%) case. The unequal distribution of bone quality among patients made it impossible to draw definite conclusions regarding the role of bone quality as a recovery predicting variable.

The two-stage traditional protocol was used in 32 (80%) cases, while in 8 (20%) cases a one-stage protocol was used. Disregarding the used protocol, the questionnaire was given only for the first surgery.

Minimal bone augmentation was required in 7 (17.5%) cases.

In 19 (48%) cases, the implants were placed in the mandible, while in 21 (52%) cases the implants were placed in the maxilla.

Thirty-three (33.7%) implants were inserted in the intercanine area and in the premolar area, respectively, and 32 (32.6%) were inserted in the molar area.

Eight (20%) patients were smokers and 5 (12.5%) patients were reported as bruxers.

**Figure 1:**
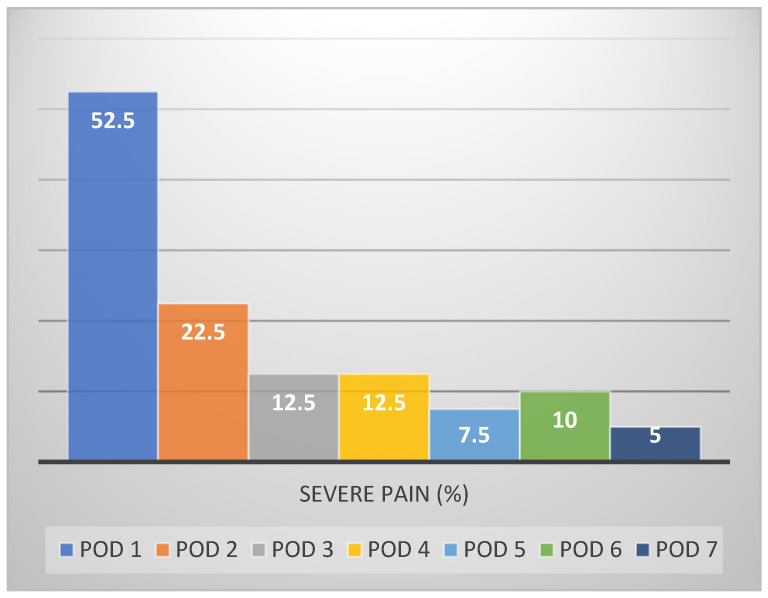
Percentage of individuals experiencing severe pain (>2) over post operative days (POD).

On postoperative day (POD) 1, 52.5% of the patients reported severe pain (score 8–10/10) at some point in the day (Figure 1), decreasing gradually by POD 2 to 22.5% and by POD 3 to 12.5%.

Consumption of analgesics also declined gradually over the first three postoperative days (80%, 45%, and 30%, respectively).

**Figure 2:**
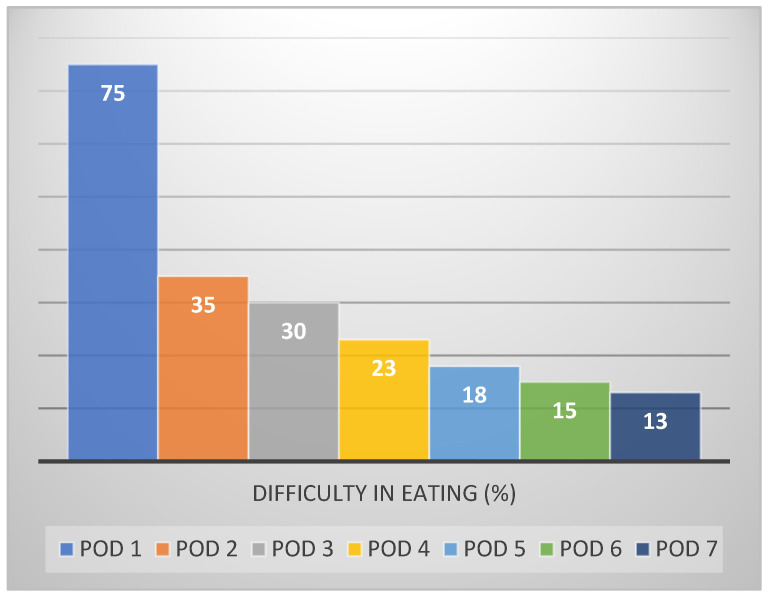
Percentage of individuals experiencing difficulty in eating (>2) over time.

On POD 1, difficulty in eating (Figure 2) was the most frequently reported feature (75%), followed by swelling (62.5%), inability to enjoy regular food (50%), substantial interference in daily activity (27.5%), and absence from work (25%).

Improvement in most oral functions (Table 1) was evident by POD 3 (inability to enjoy regular food (22.5%), swallowing (7.5%), speech (7.5%), and limitation in mouth opening (2.5%)), with the exception of difficulty in eating (23%), which improved only by POD 4.

Limitation in daily routine declined to 12.5% (four individuals) by POD 2, resembling absence from work, which reached 12.5%. Sleep was minimally affected during the entire postsurgical period.

**Figure 3:**
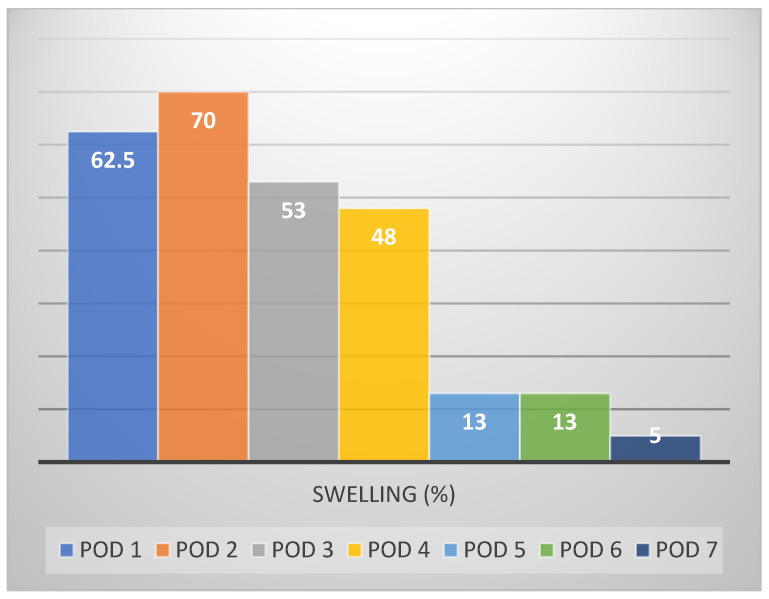
Percentage of individuals experiencing swelling (>2) over time.

Swelling, the major distressing postoperative symptom (Figure 3), resolved by POD 4–5 (13%); bleeding, food stagnation, bad taste/smell, bruising, and malaise were only marginally evident to patients in the recovery period.

The median recovery time (values of 2 or less), as reflected in swelling, required 4–5 days to reach minimal levels; the ability to eat, enjoy, and taste food along with pain and analgesics consumption required 3 days; mouth opening, speech, and everyday activity required 2 days; and within 1 day, all other measures attained minimal levels. None of the patients returned with aggravation of symptoms, for postoperative visits.

The influence of predictor variables on “recovery time” was assessed. The statistically significant predictor variables were gender and implant location. The presence of healing abutment in one-stage cases, inserting one versus several implants at the same surgical appointment, smoking, and bruxism resulted in a similar recovery period and did not can contribute to the results.

Regarding gender, women showed slower recovery regarding eating difficulties (POD 6 vs. 2, *p <* 0.05), the ability to enjoy food (POD 4 vs. 1, *p <* 0.05), everyday activity (POD 3 vs. 1, *p <* 0.05), and pain (POD 4 vs. 2, *p <* 0.05) and analgesics consumption (POD 3 vs. 1, *p <* 0.05) compared to men.

Implants placed in the anterior area of the jaw (intercanine area) showed slower recovery regarding eating difficulties compared to the posterior area (POD 6.5 vs. 3, *p <* 0.05).

## 4. Discussion

Despite progress in preoperative, operative, and postoperative management, which make dental treatment today easier than ever, a high proportion of dental patients still report concern relating to the operative and postoperative sequelae of various procedures [8,27]. Previously, pain and swelling in the first week after dental implant placement were assessed. Most patients who experienced pain reported the latter to be slight, with a peak intensity 6 h after the operation in 41.5% of cases [28]. In the present study, a daily evaluation was performed as in previous studies performed in our institution [14]. This could enable the comparison of recovery between different surgical procedures.

Local anesthetic and excessive fluid in the mouth are the most uncomfortable intraoperative experiences associated with periodontal or implant surgery [29,30,31,32]. Since the present study assessed only postoperative recovery, we did not address these issues.

Regardless of the surgical technical simplicity of dental implant placement, the obvious fact that a wound is created makes it reasonable to assume that it will have some adverse influence on several aspects of HRQOL [14,23]. Nevertheless, very few studies define the difficulties that a patient undergoing dental implant placement may expect in the immediate postoperative days.

The patient sample for the present study was young with a slight preponderance of females (65%). The results show that swelling resolved by POD 4 while the majority of the oral functions recovered within 3 days. Pain and analgesics consumption also required 3 days while all other measurements attained minimal levels within 1–2 days.

One of the limitations of the current study is the use of a single implant brand (Zimmer Biomet Dental, Palm Beach Gardens, FL, USA) only. Consequently, the influence of different implant designs on recovery was not assessed as in previous studies [33]. Correspondingly, the effect of implant geometry on pain and swelling in the first week after dental implant placement was not assessed.

Another limitation of the study is the lack of randomization. To overcome these limitations, consecutive patients were included.

The same HRQOL questionnaire was used for studying post treatment recovery [14,23]. A comparison of the present results to those previously obtained [14,23], reveal that dental implants lead to a similar postoperative recovery time (4 days). It may be speculated that, as the procedures are similar, there is a need to raise a surgical flap, perform surgery, and reach soft tissue closure by primary intention, which may be responsible for the similarity.

Gender (women) was the main predictor variable significantly affecting recovery. It affected eating difficulties, the ability to enjoy food, everyday activity, pain, and analgesics consumption. Assessment of post-surgery pain response and impairment of life activities in 42 periodontal patients between the ages 26 and 67 demonstrated that dental anxiety, fatigue, and depression were positively associated with measures of post-surgery sequelae [34]. The prevalence of the fear of dentistry among women is higher compared to men [35]. Perhaps the fact that women are more aware, more perceptive, and have a very intensive lifestyle makes their expectations to full recovery higher when compared to men. Therefore, minor interferences for the men become more noteworthy for the women.

It is perhaps surprising to see that inserting one versus several implants at the same surgical appointment resulted in a similar recovery period. Thus, the concern expressed by many patients that if several implants are performed simultaneously, the patient will suffer more, is largely unsupported.

More studies with larger sample sizes should be carried out to compare implant surgery to other oral surgical procedures. Confounding factors affecting pain perception should be considered. Tooth extraction, for example, is usually accompanied by inflammation. Post extraction pain can increase 3-fold for symptomatic teeth due to inflammatory mediators increasing nociceptors activity. Consequently, it is expected that implant placement will have less postsurgical pain and discomfort. Moreover, implant surgery is elective, and is hence more controllable than symptomatic non-elective surgical procedures. Future studies should validate such findings [7,13,25,36,37,38].

The present findings may be used as a means to provide information for the patient to evaluate, together with the more direct factors surrounding the treatment options. This study demonstrates that recovery is rapid and the practitioner should allay patient concerns surrounding the implantation procedure. This should be done with the aim of eliminating it as a factor, in favor of the objective reasons itself for or against implant placement in any given prosthetic treatment plan.

## 5. Conclusions

Within the limits of the present sample, the following may be concluded:

1. Patients should expect, in general, recovery within 4 days after dental implant placement.
2. Women will experience a delayed recovery.
3. Implants placed in the intercanine area will result in postoperative eating difficulties for nearly one week.
4. The number of implants placed during the same appointment has no effect on post treatment recovery.

## Figures and Tables

**Table 1:** Median questionnaire scores over time.

Question	Median Score						
	POD1	POD2	POD3	POD4	POD5	POD6	POD7
Has it been difficult to swallow today?	2	2	1	1	1	1	1
Has it been difficult to open your mouth today?	2	2	2	1.5	1	1	1
Were there any foods you could not eat today?	4	3	2	2	2	1.5	1
Have you enjoyed your food today?	3.5	3	2	2	2	1	1
Has speech been difficult today?	2.5	2	1	1	1	1	1
Was it difficult to sleep last night?	2	1	1	1	1	1	1
Have you missed school/work?	1.5	1	1	1	1	1	1
Has it been difficult to continue your daily activities today?	2	2	1	1	1	1	1
Has there been any swelling today?	3	3	3	2	2	1	1
Has there been bruising today?	1	1	1	1	1	1	1
Has there been bleeding today?	1	1	1	1	1	1	1
Have you felt unwell today?	1	1	1	1	1	1	1
Have you had a bad taste or bad smell in your mouth today?	2	2	1	2	1	1	1
Has there been any food debris in the operation area today?	2	1	2	1	1	1	1

## Data Availability

The data that support the findings of this study are available from the authors (A.K.) upon reasonable request.

## Appendix A

Dental implant placement questionnaire

You have just experienced a surgical procedure. In order to improve the quality of care we provide for our patients, it is important for us to know how the surgical procedure has affected you. We ask you to take a few moments to complete this survey form. Every day you will be telephoned and asked the following questions. Please choose the number that corresponds most closely to your assessment over the past 24 h.

Rate the worst pain you have felt during the past 24 h on a scale from 1 to 10 (1—not at all, 10—very much).

Have you taken any medication to relieve pain today? (no = 0, yes = 1)

For the following questions, please use this system:

Not at all = 1, Very little = 2, A little = 3, Quite a lot = 4, Very much = 5

Has it been difficult to swallow today?

Has it been difficult to open your mouth today?

Were there any foods you could not eat today?

Have you enjoyed your food today?

Has speech been difficult today?

Was it difficult to sleep last night?

Have you missed school/work?

Has it been difficult to continue your daily activities today?

Has there been any swelling today?

Has there been bruising today?

Has there been bleeding today?

Have you felt unwell today?

Have you had a bad taste or bad smell in your mouth today?

Has there been any food debris in the operation area today?
